# Efficacy and Safety of Combined Platelet‐Rich Plasma With Fractional Laser for Adult Patients With Vitiligo: A Systematic Review and Meta‐Analysis of Randomized Controlled Trials

**DOI:** 10.1111/jocd.70245

**Published:** 2025-05-19

**Authors:** Xiaowei Feng, Shali Jiang, Xingwei Zou, Yongqiong Deng, Jinwei Xie

**Affiliations:** ^1^ Department of Dermatovenerology Chengdu First People's Hospital Chengdu China; ^2^ Department of Orthopedics Surgery West China Hospital of Sichuan University Chengdu China

**Keywords:** fractional laser, meta‐analysis, platelet‐rich plasma, repigmentation, vitiligo

## Abstract

**Objective:**

Platelet‐rich plasma (PRP) is a novel treatment option for vitiligo. It has been reported to be effective in combination with other methods, such as fractional laser. However, there is no consensus on the specific combined use of PRP with fractional laser. Therefore, this meta‐analysis assessed the efficacy and safety of this combination regimen compared to control groups for vitiligo.

**Methods:**

A comprehensive literature search was conducted for relevant randomized controlled trials comparing PRP plus fractional laser with other routine treatments published from inception to January 2025. Data regarding the mean grade of repigmentation, patient's satisfaction score, rate of satisfactory repigmentation or no response, and incidence of side effects were extracted and meta‐analyzed using a fixed or randomized model.

**Results:**

Seven studies involving 366 patients were reviewed. The combination of PRP and fractional laser therapy significantly improved the mean grade of repigmentation (mean difference [MD] = 1.58; 95% confidence interval [CI] = 1.08–2.07; *p* < 0.01) and patients' satisfaction score (MD = 1.87; 95% CI = 0.90–2.83; *p* = 0.0001), while also reducing the no response rate (risk ratio [RR] = 0.54; 95% CI = 0.32–0.92; *p* = 0.02), compared with control groups, including monotherapy, topical drug or sun exposure only, and fractional lasers plus narrowband ultraviolet B. Additionally, the incidence of adverse events for combination therapy was comparable to that of control groups (RR = 0.86; 95% CI = 0.69–1.07; *p* = 0.17).

**Conclusions:**

This meta‐analysis provides evidence supporting the combined use of PRP and fractional laser therapy as a valuable and safe treatment modality for patients with vitiligo, based on its superiority to control groups and comparable rates of side effects. However, more well‐designed and large‐scale studies are required to confirm it.

## 1 Introduction

Vitiligo represents an acquired hypopigmentation condition, manifested through white macules and patches due to the lack of functional melanocytes [1]. Even though it does not pose a life‐threatening risk, its long‐lasting nature can substantially impact patients' esthetic appearance and psychosocial well‐being. Regarding the pathogenesis of vitiligo, several key hypotheses exist, such as the genetic, environmental, neural, autoimmune, biochemical, and melanocytorrhagy theories [2]. As a result, the treatment options for vitiligo are varied, ranging from topical and systemic medications, phototherapy, laser treatment, surgical procedures, and even the application of cosmetics [3, 4]. Nevertheless, none of these treatment methods yield highly encouraging outcomes, particularly when dealing with certain localized, stable, and treatment‐resistant lesions. Accordingly, novel therapeutic approaches have come to the fore. These include fractional laser and platelet‐rich plasma (PRP), both of which have been recognized as effective in the treatment of vitiligo.

Fractional laser is widely applied in dermatological and cosmetic clinics. It includes ablative fractional laser and nonablative fractional laser; the former includes carbon dioxide (CO_2_) fractional laser and Er: yttrium–aluminum–garnet fractional laser, and the latter includes 1565 and 1927 nm lasers. In particular, ablative segmented lasers are widely used due to their potential immune effect [5], which several studies have verified as a promising supplementation method for treating vitiligo [6, 7]. The underlying mechanism might involve immediate tissue constriction, a decrease in the size of vitiligo lesions, and an augmented secretion of cytokines and diverse growth factors during the wound‐healing procedures that are essential for pigmentation. Additionally, fractional lasers have the capacity to boost the transdermal permeation of ultraviolet light and local medications, thus enhancing their effectiveness [8].

PRP represents an autologous concentrate of platelets within plasma. It is distinguished by the existence of numerous growth factors [9]. These growth factors are recognized for their role in regulating multiple processes, such as cell migration, adhesion, growth, differentiation, and maturation [10]. As a crucial element in regenerative medicine, PRP shows significant potential for treating various diseases and injuries, including vitiligo [11]. Numerous clinical trials and reviews have explored the efficacy of PRP when used in conjunction with other approaches, like narrowband ultraviolet B (NB‐UVB), the 308 excimer laser, and surgical treatment [12, 13, 14, 15, 16]. These studies suggest that combined treatment can improve results and decrease the incidence of adverse events. However, up to now, large‐scale trials are in short supply, and there is no unified conclusion regarding the effectiveness and safety of the combination of PRP and fractional lasers for treating vitiligo. To fill this void, this systematic review and meta‐analysis were carried out.

## 2 Materials and Methods

### 2.1 Literature Search

In accordance with the PRISMA guidelines [17], an exhaustive search was carried out. The electronic databases utilized were PubMed, EMBASE, Cochrane Central Register of Controlled Trials, and Web of Science, spanning from their inception until January 1, 2025. The search terms consisted of “vitiligo,” “platelet‐rich plasma,” “platelet concentrates,” “PRP,” “laser,” and “fractional laser,” with no language limitations imposed. Moreover, the reference lists of the cited studies were examined for eligibility. Two reviewers evaluated all relevant studies independently.

### 2.2 Study Selection

Studies were chosen according to the following inclusion criteria: (1) randomized controlled trials (RCTs); (2) patients diagnosed with vitiligo; (3) the experimental group was treated with PRP combined with fractional lasers, regardless of control group treatments; and (4) at least one primary endpoint of interest had to be reported. Studies were excluded under the following circumstances: (1) duplicate publications, (2) nonrandomized clinical trials, and (3) studies with inappropriate outcomes or unavailable full text, even after contacting the author. Any discrepancies between the reviewers were settled through discussion and reached a consensus.

### 2.3 Data Extraction and Quality Assessment

Two reviewers separately extracted data from eligible studies, including details of the first author, publication year, number of patients in both the experimental and control groups, patients' characteristics such as age and sex, specific details about the interventions and comparisons, number of treatment sessions, and the length of follow‐up period. In the analysis, the outcomes of concern were as follows: the average improvement grade of repigmentation after treatment, evaluated on a four‐point scale (Grade 4 = excellent for 75%–100% repigmentation, Grade 3 = good for 50%–75%, Grade 2 = moderate for 25%–50%, and Grade 1 = mild for < 25%), the patients' satisfaction degree measured by a 10‐point visual analog scale (VAS), the rate of poor or satisfactory repigmentation based on the Physician's Global Assessment (PGA, poor: no repigmentation, satisfactory: < 25%, good: 25%–50%, very good: 50%–75%, and excellent: > 75%), and the incidence of side effects. Data extraction followed the intention‐to‐treat principles using a preestablished form.

The quality was independently evaluated by two authors using the Cochrane Collaboration's risk‐of‐bias instrument in randomized trials and was scored using the modified Jadad scale. Studies scoring ≥ 4 (out of 8) were considered high quality. In case of any disagreements, a third reviewer was involved to reach a resolution.

### 2.4 Statistical Analysis

The systematic review, along with meta‐analysis, was conducted utilizing Review Manager (RevMan, version 5.4, the Cochrane Collaboration, 2020). Data preprocessing was performed in Microsoft Excel. The mean grade of repigmentation and patients' satisfaction score were analyzed using the mean difference (MD) and 95% confidence interval (CI). The risk ratio (RR) and 95% CI were used in analyzing dichotomous outcomes (rates of different repigmentation and incidence of adverse events). Statistical heterogeneity was tested using the Chi‐squared test or I^2^ statistic (*I* ^*2*^ > 50% or *p* < 0.10). When there was no evidence of heterogeneity, a fixed‐effect model was employed to estimate the effect. Otherwise, a random‐effects model was implemented instead. Subgroup analyses were conducted based on the different control groups or side effects. Sensitivity analysis was conducted to evaluate the impact of each individual study by excluding one study at a time. Funnel plots were used to assess publication bias, with *p* < 0.05 indicating statistical significance.

## 3 Results

### 3.1 Study Information and Participant Characteristics

**FIGURE 1:**
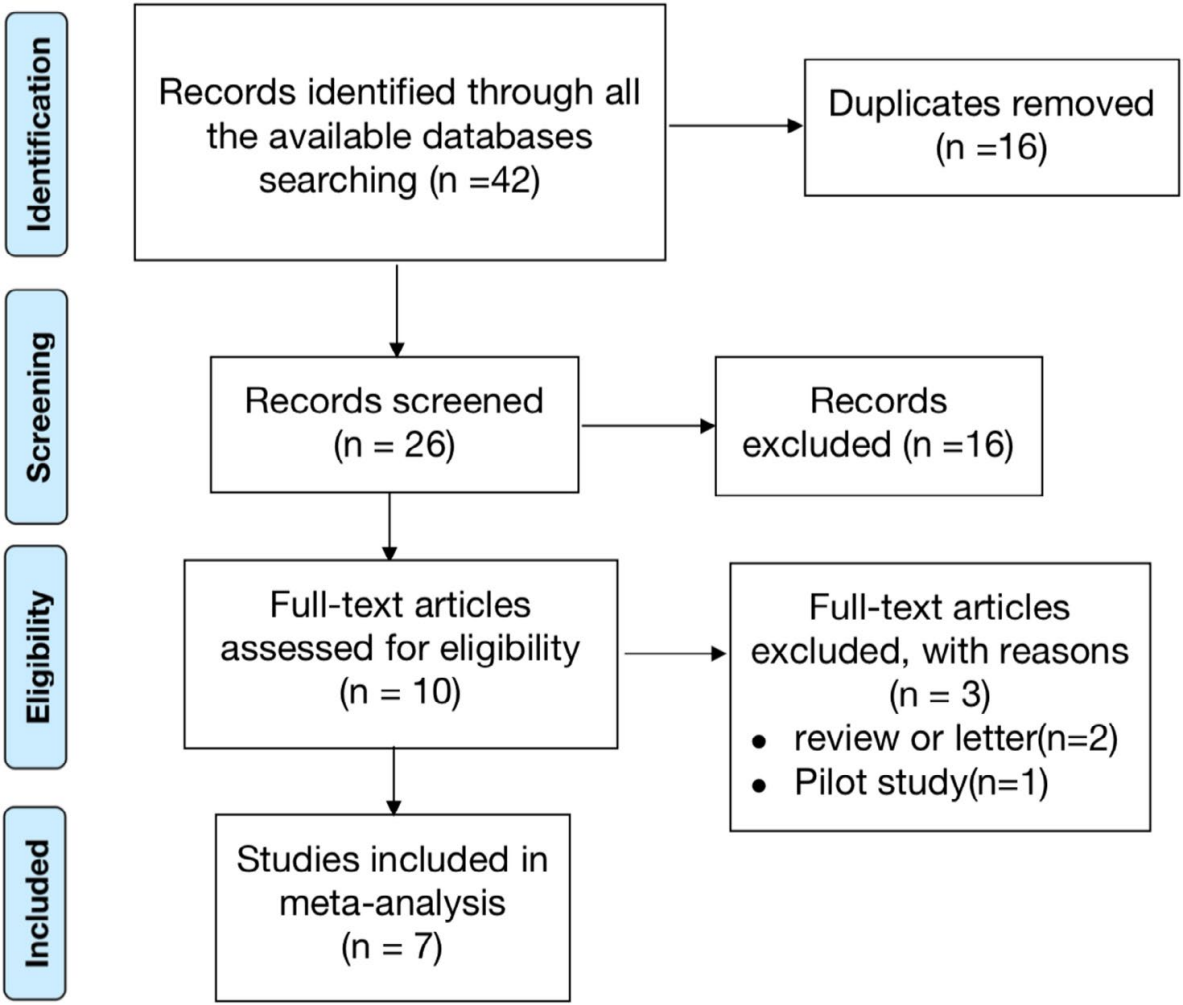
Flowchart of the study selection process.

In the process of study retrieval, a total of 42 studies were initially obtained, and after removing duplicates (*n* = 16) and screening titles/abstracts (*n* = 16), 10 studies were finally reviewed. After reviewing the full text, three more studies were excluded, including one pilot study, one letter, and one review article. Eventually, seven articles [18, 19, 20, 21, 22, 23, 24] published between 2017 and 2024, with 348 patients (135 men and 213 women), were included in the meta‐analysis (Figure 1). Detailed information about the included studies is presented in Table 1.

**TABLE 1:** Characteristics of included studies.

Studies	Study design	Sample (E/C)	Age (yrs)	Gender (F/M)	Interventions		PRP preparation	Laser protocol	Treatment sessions/duration	Side effects
					Experiment group (E)	Control group (C)				
Abdelghani 2017 [18] (Egypt)	Prospective randomized parallel group comparative	20/20/20/20	E: 33.9 ± 11.889; C: 29.6 ± 10.802; 34.9 ± 15.386; 36.95 ± 13.04	50/30	FCO_2_ + PRP	FCO_2_ + NB‐UVB; FCO_2_ alone; PRP alone	10–20 mL blood; double centrifugation, first 252 g for 10 min, then 448 g for 10 min	Power 6 W, dot mode; spacing 550 μm; dwell time 400 μs; scanning mode; smart track; single stack; square shape; ratio 10/10; size 100%	4 biweekly sessions/5 months	Erythema
Kadry 2018 [19] (Egypt)	Prospective randomized intrapatient comparative controlled	30/30/30/30	32.03 ± 12.29	22/8	FCO_2_ + PRP	FCO_2_ alone; PRP alone; sun expose	8 mL blood; centrifuged at 1500 rpm for 5 min	KES, Beijing, China; minimum energy (30–50 mJ); Scanner spot; density 0.6; static mode; two passes with minimal overlap; over a thin rim of healthy skin (2 mm)	6 biweekly sessions/6 months	Pain; hyperpigmentation; erythema
Hamid 2024 [20] (Egypt)	Randomized blinded clinical	20/20	E: 25.90 ± 2.5; C: 26.00 ± 2.6	27/13	Fractional erbium–YAG laser + PRP	Microneedling with PRP	First centrifuged at 3000 rpm for 7 min; second at 4000 rpm for 5 min; PRP rose to the top	Fractional Er:YAG laser; FotonaXs Dynamis, Slovenia; short pulse mode; 1400 mJ; pixel; spot size 7 mm	12 biweekly sessions/6 months	Erythema; burning; pain
Raizada 2021 [21] (India)	Prospective randomized comparative open‐label interventional	30/28	E:31.43 ± 8.19; C:32.17 ± 8.63	36/22	FCO_2_ + PRP	FCO_2_ alone	20 ml blood; first centrifuged at 1500 rpm for 10 min; REMI Elektrotechnik, Korea; second 3000 rpm for 10 min; mixed into 2 mL of plasma	30 W; FIRE XEL from Bison Medical, Korean; 1 mm density, 1500 μs pulse width; 3000 ms repeat delay; 9 times overlap; 202.5 mJ energy	1 treatment session/3 months	Pain; burning; erythema; crust
Ahlawat 2023 [22] (India)	Prospective comparative interventional	30/30	E: 27.46 ± 8.91; C: 29.86 ± 11.1	34/26	FCO_2_ + PRP	FCO_2_ alone	Intradermal injections of autologous PRP once a month after all aseptic precautions	Point energy 70–100 MJ; 20 W; duration 5 ms; interval 4 ms; distance 0.7 mm; scan mode; three times	4 monthly sessions/5 months	Erythema; burning; pain; hyperpigmentation
Afify 2020 [23] (Egypt)	Self‐controlled randomized clinical	20/20/20/20/20	46.5 ± 14.5	11/9	FCO_2_ + PRP	FCO_2_ alone; PRP alone; FCO_2_ + NB‐UVB; FCO_2_ + NB‐UVB + PRP	10 mL venous blood; Scientific System, China; first centrifugation 160 g (1000 rpm) for 10 min; second 400 g (2000 rpm) for 10 min.	15 W; pulse width 1 ms; overlap 1 time; square shape; density 0.5; energy/dot 15 mJ.	4 biweekly sessions/3 months	Pain; itching; expansion; erythema; crust
Omar 2024 [24] (Egypt)	Prospective randomized parallel group comparative	20/20/20	E: 30.80 ± 8.21; C: 32.47 ± 5.69; 33.60 ± 5.75	33/27	FCO_2_ + PRP + latanoprost	Latanoprost alone; FCO_2_ + latanoprost	20 mL blood; centrifuged at 1500 rpm for 10 min; REMI Elektrotechnik, Korea; then supernatant centrifuged at 3000 rpm for 10 min; mixed into 2 mL plasma	10 600, KESA, Beijing, China; minimum energy (30–50 mJ); scanner spot; density 0.6; static mode; two passes with minimal overlap; over a thin rim of healthy skin (2 mm).	6 biweekly sessions/4 months	Erythema; crust; pain

Abbreviations: C, Control group; E, experimental group; F, female; FCO2, fractional CO2 laser; M, male; PRP: platelet‐rich plasma.

### 3.2 Quality Assessment of the Included Studies

**FIGURE 2:**
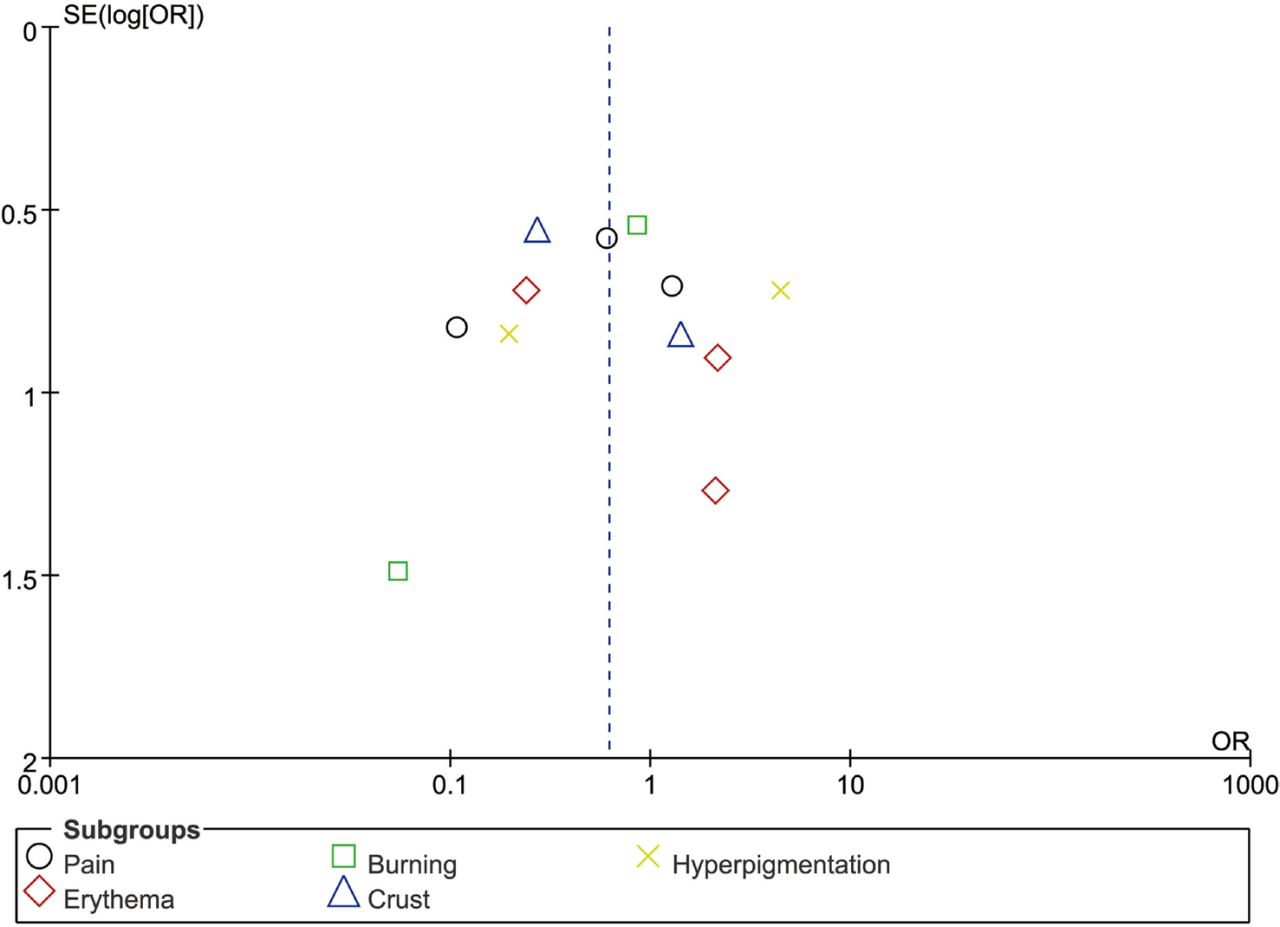
Funnel plot of bias risk.

Results on the methodological quality and bias risk of the included studies are summarized in Table 2. All RCTs scored ≥ 4 on the modified Jadad scale, indicating high quality, while funnel plots revealed low publication bias (Figure 2).

**TABLE 2:** Quality assessment of randomized controlled studies according to the Cochrane Risk‐of‐Bias Tool (“Unclear, lack of information or uncertainty over the potential bias”).

Studies	Random sequence generation	Allocation concealment	Blinding	Incomplete outcome data	Selective reporting	Other bias	Jadad score (0–8)
Abdelghani 2017 [18]	Low	Unclear	Unclear	Low	Low	Low	5
Kadry 2018 [19]	Low	Unclear	Unclear	Low	Low	Low	6
Hamid 2024 [20]	Low	Low	Low	Low	Low	Low	8
Raizada 2021 [21]	Low	Unclear	High	Low	Low	Low	5
Ahlawat 2023 [22]	Unclear	Unclear	Unclear	Low	Low	Low	4
Afify 2020 [23]	Low	Low	Low	Low	Low	Low	8
Omar 2024 [24]	Low	Unclear	Unclear	Low	Low	Low	7

### 3.3 Effects of PRP Plus Fractional Lasers Compared With Control Groups

#### 3.3.1 Mean Grade of Repigmentation

**FIGURE 3:**
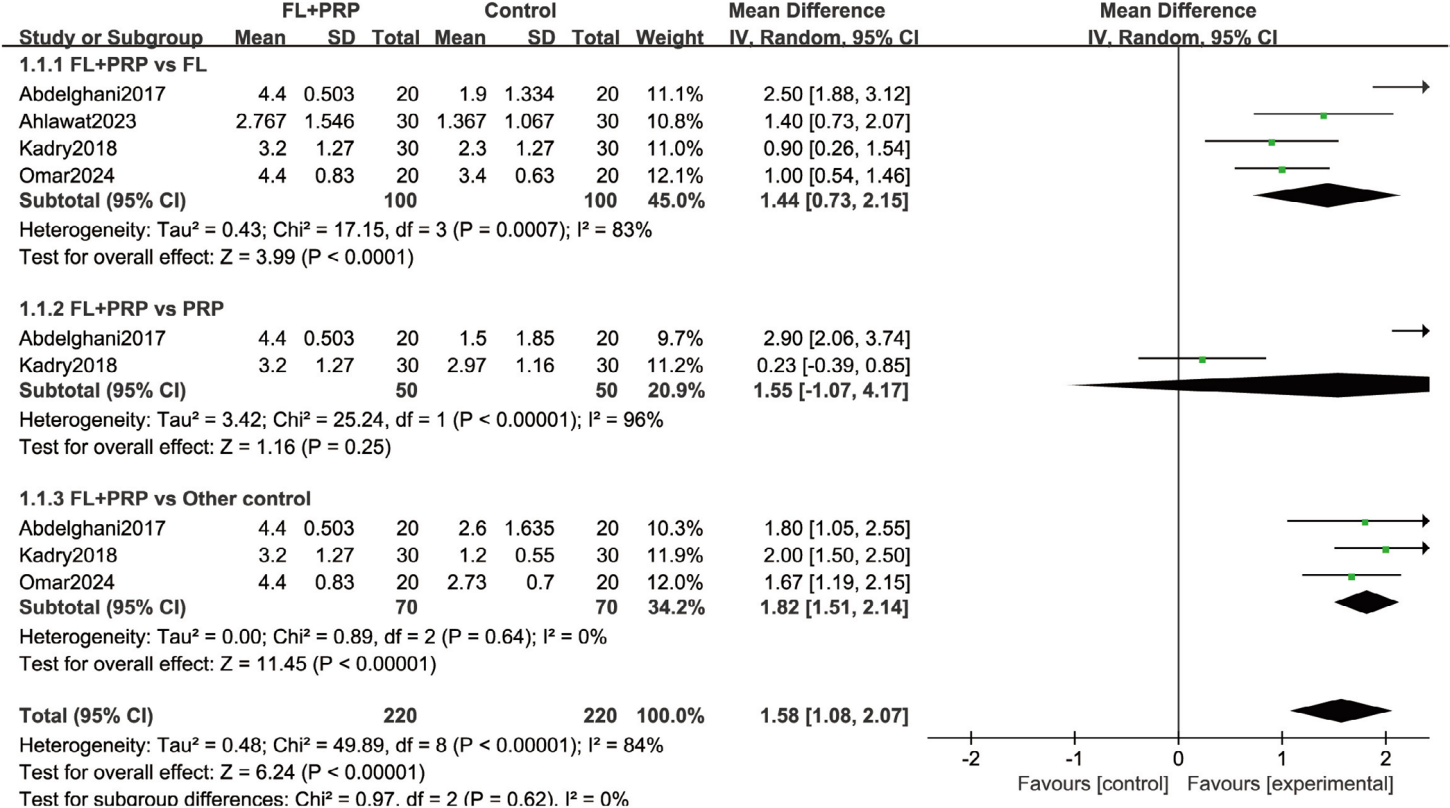
Mean grade of repigmentation at the end. FL, Fractional laser; PRP, platelet‐rich plasma.

Four studies [18, 19, 22, 24] involving 320 cases reported the mean improvement grade for repigmentation at the final follow‐up. Pooled analysis, conducted using a random‐effects model due to substantial heterogeneity, indicated a significantly higher mean improvement grade in the experimental group compared to the control group (MD = 1.58; 95% CI = 1.08–2.07; *p* < 0.01). Subgroup analysis revealed significantly higher grades in the experimental group than the fractional laser alone (MD = 1.44; 95% CI = 0.73–2.15; *p* < 0.01) and other control groups (MD = 1.82; 95% CI = 1.51–2.14; *p* < 0.01), including sun exposure only, latanoprost use alone, and fractional laser combined with NB‐UVB groups. However, no significant grade was observed when the combination group was compared with PRP alone (MD = 1.55; 95% CI = −1.07 to 4.17; *p* = 0.25; Figure 3).

#### 3.3.2 Patients' Satisfaction Score

**FIGURE 4:**
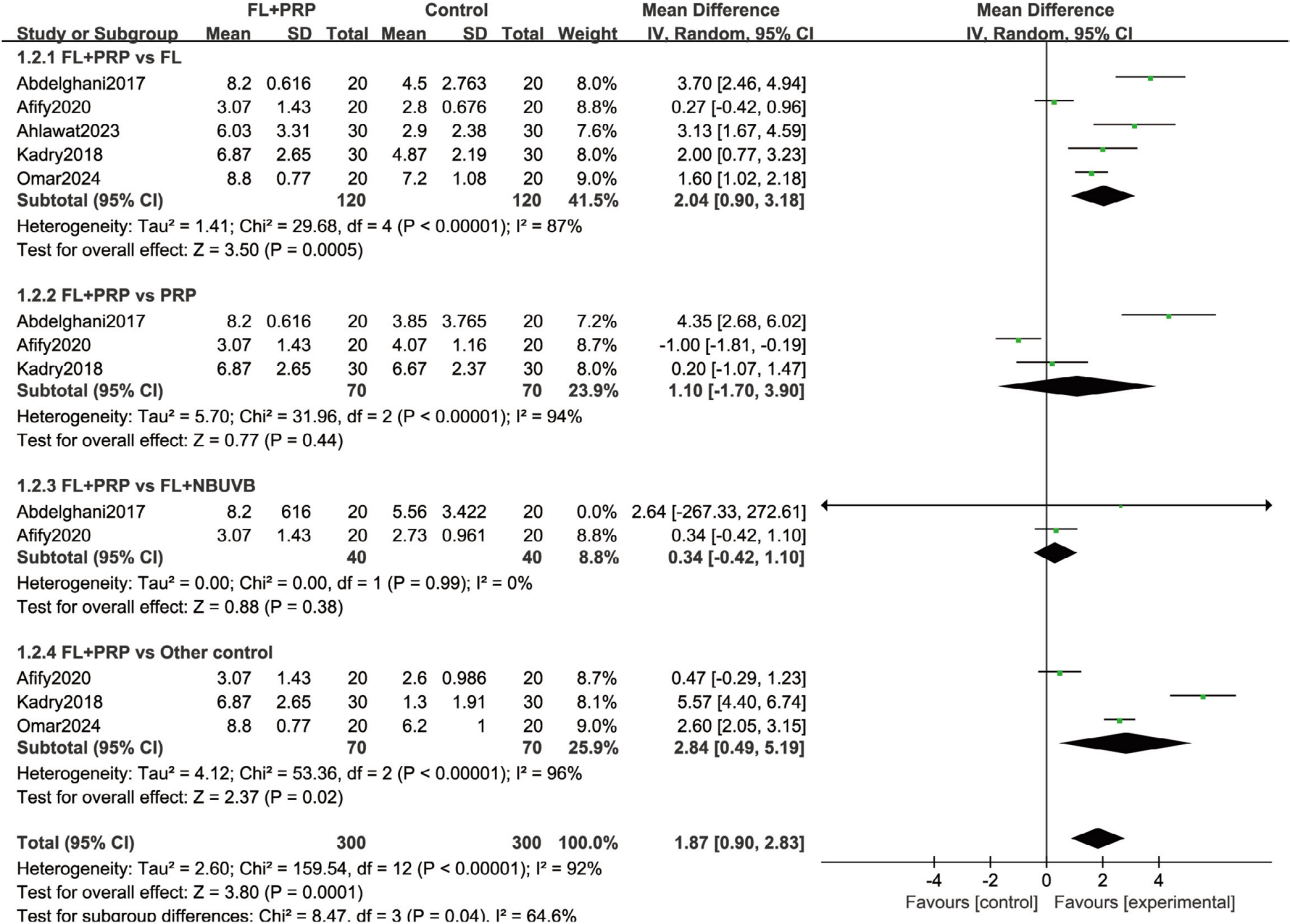
Patients' satisfaction scores. FL, Fractional laser; PRP, platelet‐rich plasma.

Five studies [18, 19, 22, 23, 24] involving 420 cases documented the degree of patients' satisfaction between the two groups. Quantitative synthesis indicated a significantly higher level of patient satisfaction in the experimental group than in the control group (MD = 1.87; 95% CI = 0.90–2.83; *p* = 0.0001). A random‐effects model was applied due to high heterogeneity, and the subgroup analysis revealed significantly higher patients' satisfaction compared to fractional laser alone (MD = 2.04; 95% CI = 0.90–3.18; *p* = 0.0005) and the other control groups (MD = 2.84; 95% CI = 0.49–5.19; *p* = 0.02). However, no significant difference was observed when compared with the PRP alone group (MD = 1.10; 95% CI = −1.70 to 3.90; *p* = 0.44) and fractional laser plus NB‐UVB group (MD = 0.34; 95% CI = −0.42 to 1.10; *p* = 0.38; Figure 4).

#### 3.3.3 Rates of Different Responses

**FIGURE 5:**
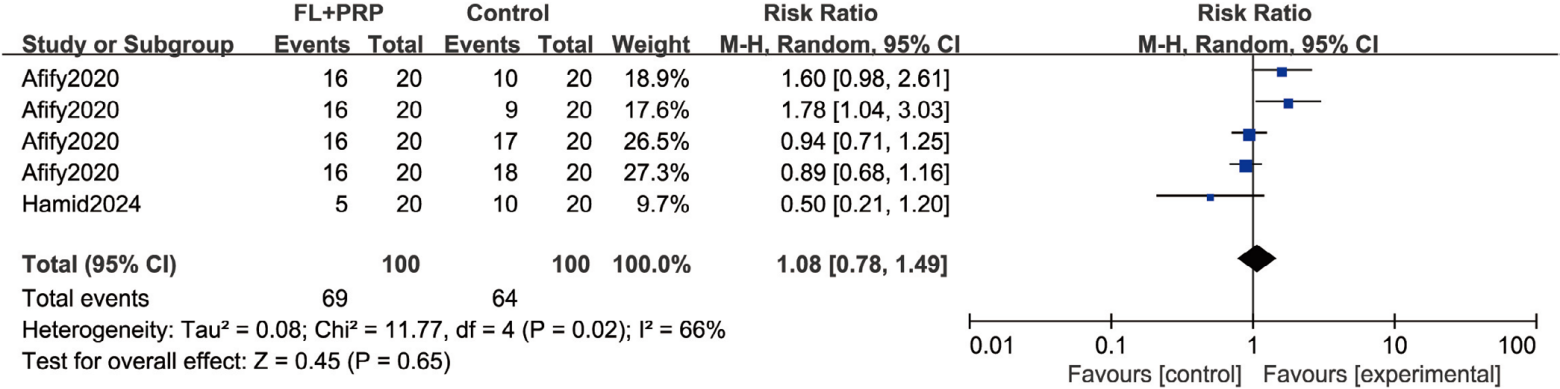
Response rate of satisfactory repigmentation. FL, Fractional laser; PRP, platelet‐rich plasma.

**FIGURE 6:**
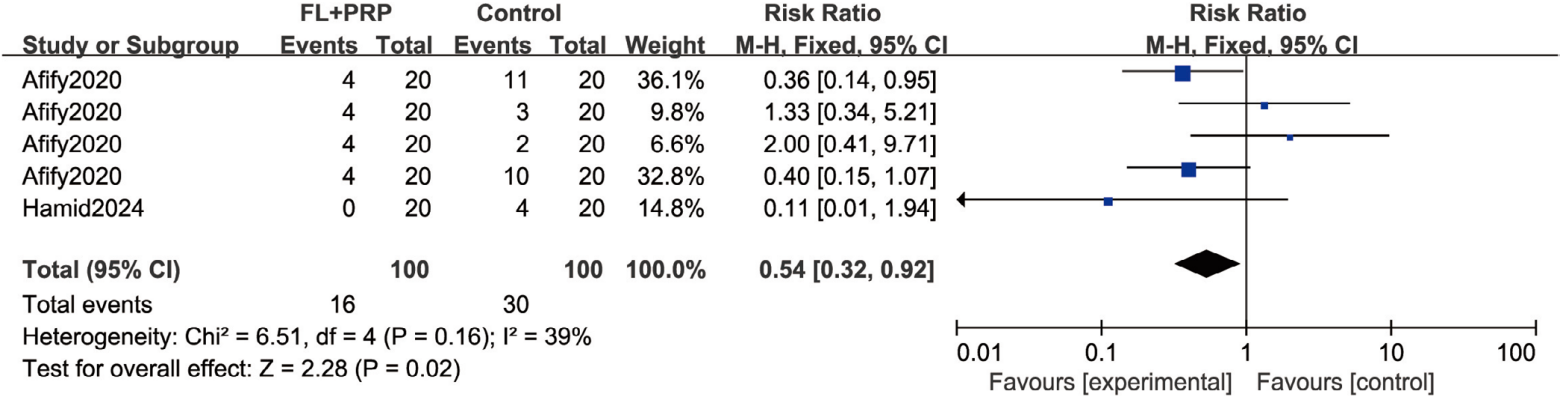
Response rate of poor repigmentation. FL, Fractional laser; PRP, platelet‐rich plasma.

Two studies [20, 23] involving 140 patients evaluated the response rates for different grades of repigmentation. Since the control group treatments included microneedling with PRP, fractional laser, PRP injection, fractional laser plus NB‐UVB, and FL + NB‐UVB + PRP, the two studies were divided into four comparisons. Quantitative analysis, using a random‐effects model, indicated a similar response rate for satisfactory repigmentation between the experimental and control groups (RR = 1.08; 95% CI = 0.78–1.49; *p* = 0.65; Figure 5). However, the experimental group presented a significantly lower rate of poor repigmentation (RR = 0.54; 95% CI = 0.32–0.92; *p* = 0.02) based on a fixed‐effects model due to low heterogeneity (Figure 6).

### 3.4 Side Effects and Adverse Events

**FIGURE 7:**
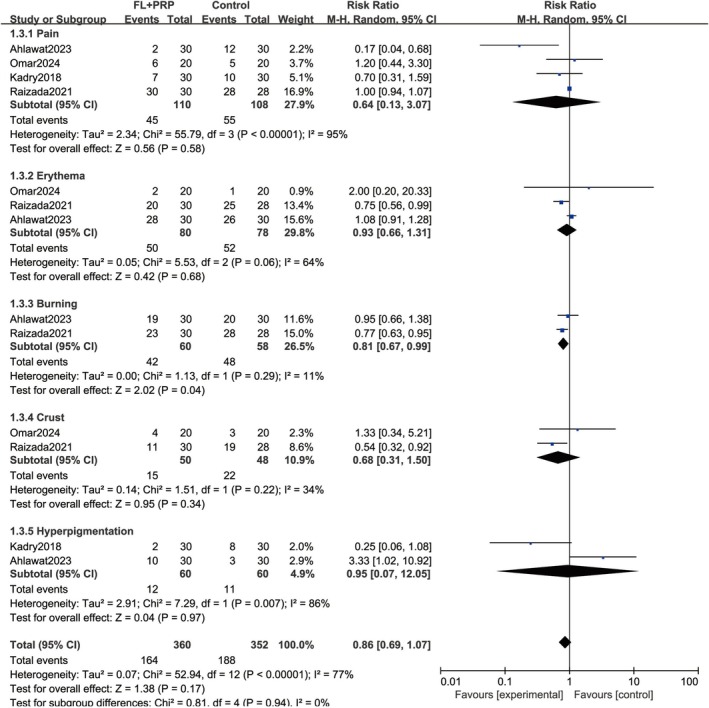
Rates of side effects. FL, Fractional laser; PRP, platelet‐rich plasma.

All included studies documented some side effects and adverse events, mainly including mild, tolerable pain, erythema, burning, crust, and hyperpigmentation, which all could be resolved without special treatment in a short time. Pooled analysis revealed a nonsignificant difference in the incidence of side effects between the fractional lasers combined with PRP and control groups (RR = 0.86; 95% CI = 0.69–1.07; *p* = 0.17). Similar results were observed in a subgroup analysis of different side effects (Figure 7).

### 3.5 Sensitivity Analysis

The sensitivity analyses were conducted privately, revealing that none of the studies qualitatively altered the pooled results, confirming the robustness of the findings.

## 4 Discussion

This meta‐analysis is the first to comprehensively evaluate PRP combined with fractional lasers for vitiligo treatment compared to routine therapies. Despite limited trials and small sample sizes, the selected high‐quality studies highlight the potential benefits of this novel combination therapy.

Vitiligo affects approximately 0.36% of the global population, with a higher prevalence in adults (0.67%) than in children (0.24%) across all regions [25], especially in transplant recipients [26]. The incidence remains relatively stable and increases slightly every year. Asian populations are disproportionately affected, with a lifetime risk of 1 in 28 compared to 1 in 109 globally [27]. Common comorbidities associated with vitiligo are diabetes, eczema, thyroid disease, and rheumatoid arthritis [28], with an increased risk of cardiovascular diseases, such as cerebral infarction and venous thromboembolism, compared to healthy individuals [29]. Interestingly, the cancer incidence rates in patients with vitiligo are not elevated and may even reduce the risk of melanoma, lung cancer, and bladder cancer [30]. While vitiligo may be overlooked due to its non–life‐threatening nature, it may impact mental health, potentially leading to anxiety, depression, and sleep disturbance. These effects may be more common in White individuals than in Black and Asian populations [28]. These findings highlight the urgent need for effective vitiligo treatments.

Vitiligo pathogenesis is complex and multifactorial, involving genetic predisposition, epigenetic influences, and critical biochemical and molecular pathways that play pivotal roles in its development [31]. Diverse treatment strategies have been employed, including drugs, phototherapy, surgical techniques, lasers, and some regenerative medicines. However, none of these treatments has emerged as a gold standard. This variability has led to the inclusion of diverse control groups across trials, even within a single trial, resulting in the repeated and reasonable use of experimental group data for independent comparisons. Subgroup analyses were performed to minimize discrepancies; however, the overall effect should still be interpreted cautiously, demonstrating a preference for the experimental combination group. These results align with the other two meta‐analyses, which studied the effect of PRP combined with excimer laser or some other methods [14, 32]. However, whether the observed benefits are truly attributable to laser, PRP, or the synergistic effect of multiple interventions is questionable. In the future, more large‐scale and long‐term follow‐up RCTs that put different treatment groups, including a laser‐only group, a PRP‐only group, and a combination of the laser and PRP group in comparison, are needed.

Concerning the experimental group, among the seven included trials, six used CO_2_ lasers as fractional lasers, while only one [20] employed the fractional erbium–YAG laser due to their lower thermal damage and faster healing properties [33]. Both are categorized as ablative fractional lasers, known for their ability to disrupt the skin barrier, release cytokines and growth factors, stimulate melanocytes, and enhance the penetration of topical drugs [34], ultimately enhancing the effects of other methods alone, as indicated in this meta‐analysis. A comparative study by El‐Mongy et al. *[* 35] found that combining fractional laser with PRP demonstrated better improvement than PRP alone. Different results were observed by Kadry et al. *[* 19], who indicated that the combination group was more effective than using PRP alone, although the difference was nonsignificant. However, the study by El‐Mongy et al. *[* 35] was a letter to the editor with no available data for inclusion in the current meta‐analysis. With regard to specific laser parameters, there is currently no unified standard yet. Commonly, an initial test dose of laser is administered, then the minimum energy level (ranging from 30 to 50 mJ) that can cause erythema is selected. The scanner spot is modified according to the size of the lesion, with a density of 0.6 in the static mode. The laser is applied in two passes with minimal overlapping and used on the vitiligo‐affected area as well as a narrow border (2 mm) of the adjacent healthy skin [19]. More trials exploring the optimal laser parameters and administration are needed.

On the other hand, PRP is a rich source of cytokines and growth factors, which are vital in modulating tissue repair, promoting cellular proliferation, and stimulating melanocyte proliferation and repigmentation in vitiligo lesions [36, 37]. Previous meta‐analyses, including those by Wang Z et al. *[* 38] and Jafarzadeh et al. [39], highlighted the effectiveness and safety of PRP as a potential supplementary therapeutic measure or an alternative treatment in chronic wounds and vitiligo lesions. To date, no systematic review has explored the efficacy and safety of combined therapy of PRP with fractional laser, making this analysis a novel contribution. Nonetheless, there is no consensus on the optimal PRP preparation protocols and standardized operational modes; most studies, including the literature we have incorporated, adopt a double‐centrifugation (first to isolate plasma without spinning down platelets and second to separate platelets from platelet‐poor plasma) protocol, which has a higher platelet concentration than single centrifugation [18]. Intradermal injection using a 30G needle was intralesional and 1 cm perilesional to stimulate the proliferation of surrounding melanocytes via the released growth factors. Each injection was 0.1 mL with a maximum of 1–2 mL/session in volume, with 0.5–1 cm spacing between injection sites [23]. A platelet concentration exceeding 1 million/L (approximately four to seven times the average levels) was considered an indication of a successful preparation and was assumed to be therapeutically effective.

The mean grade of repigmentation and patients' satisfaction score were the primary outcomes in this meta‐analysis. These measures were selected as they are commonly evaluated in most trials and represent different aspects of the therapeutic effect, which may not align with each other. Specifically, the mean grade of repigmentation is an objective measure, while the patients' satisfaction score is a subjective measure. The patients' satisfaction is related to several aspects, including the instant effect, uncomfortable feeling, cost, and the treatment course [40]. The experimental combination group, which is usually conducted biweekly, promotes good compliance, and PRP injection is supposed to aid in healing injuries caused by fractional lasers and alleviate the side effects due to their anti‐inflammatory effect. Consequently, this combination group not only guarantees a better therapeutic effect but also a higher patients' satisfaction score. However, the difference in the mean grade of repigmentation in the subgroup of combined therapy versus PRP monotherapy was statistically nonsignificant (MD = 1.55; 95% CI = −1.07 to 4.17; *p* = 0.25). This could be attributed to an insufficient number of studies or the dominant role of PRP in combination therapy. As for monotherapy comparison, in the study by Abdelghani et al. *[* 18], the mean grade of repigmentation was 1.5 in the PRP group and 1.9 in the laser group; and the mean satisfaction VAS was 3.85 in the PRP group and 4.5 in the laser group. Conversely, Afify et al. *[* 23] revealed that the mean satisfaction VAS in the PRP group was higher than that in the Laser group (4.1 ± 1.2 vs. 2.8 ± 0.7), while the percentage of Grades 0 and 1 repigmentation was similar between the two groups (90% vs. 90%). So, more studies are needed to figure out which monotherapy is more effective.

The rates of satisfactory and poor repigmentation were also pooled in this meta‐analysis. While the rate of satisfactory repigmentation was not significantly different, the rate of no response was significantly lower in the experimental group, indicating that the fractional laser combined with PRP can promote each other's effects and response rate in vitiligo. However, these findings should be interpreted cautiously, as limited trials could be included for subgroup analysis.

Other outcomes, such as the Vitiligo Area Scoring Index (VASI) and histopathological change, have also been used to assess changes in lesions in vitiligo research and clinical practice. However, few studies have reported these outcomes, making it challenging to analyze the overall effect of combined therapy. Two studies [19, 24] that adopted histopathological change as an index revealed that HMB‐45 turned out positive in perilesional cells after treatments and was significantly higher in the combination therapy group. Regarding the VASI score, one study [21] demonstrated a significantly greater reduction in the experimental combination group compared to the fractional laser alone at each subsequent follow‐up visit. Conversely, a study by Hamid et al. *[* 20] revealed that both the fractional laser plus PRP group and microneedling plus PRP group exhibited a statistically significant decrease in VASI scores compared to the baseline; however, no significant difference was observed between the two combination groups. Therefore, future studies focusing on these indicators are needed.

Regarding safety, our systematic review demonstrated that the combination therapy was well‐tolerated, with only a small proportion of patients experiencing mild adverse events, such as erythema and pain, in a short time. Other complications, including burning, crust, and hyperpigmentation, primarily attributed to laser, were also observed. The incidence rate of adverse events was comparable between the combination therapy and control groups, and no serious adverse reactions were reported that would affect the progress of the included trials. However, the overall incidence of side effects was approximately 50% (46% in the experimental group and 50% in the control group), leading to temporary discomfort for patients. Further studies with larger sample sizes are warranted to improve the treatment and enhance patients' experience.

In summary, the combination of fractional laser and PRP has been identified to have comprehensive clinical benefits. First of all, fractional laser can create microchannels on the skin surface, stimulate the skin's self‐repair mechanism, and promote various growth factors in PRP to act on melanocytes, accelerating their proliferation and migration to the vitiligo‐affected area, thereby promoting pigment restoration. In the second place, the combination can improve blood circulation and the microenvironment of the skin in the vitiligo area, providing more favorable conditions for the growth and survival of melanocytes and improving the treatment effect. In the third place, compared with some traditional treatment methods for vitiligo, such as long‐term topical medications or phototherapy, the treatment course of fractional laser combined with PRP is relatively short, and the effect is more significant, which helps to improve patient compliance. Last but not least, PRP can relieve the adverse reactions like skin redness and pain caused by laser and accelerate recovery. Some other measures, including postoperative cold compress, antibiotic ointment or growth factor gel, sun protection, healthy lifestyle, and close follow‐up, also have great significance for boosting clinical efficacy and decreasing side reactions.

This systematic review and meta‐analysis have some limitations. First, as PRP application for vitiligo treatment is relatively new, only a few eligible RCTs could be included in the pooled analysis, especially for subgroup analysis. Second, some eligible studies lacked a clear description of patient blinding and the allocation concealment process, which may introduce the potential risk of bias. Third, all included trials were either from Egypt or India, with varying clinical characteristics such as the anatomical sites of vitiligo lesions, lesion size, and duration, which may have affected the results of each trial, thereby reducing the overall robustness of our meta‐analysis conclusion. More studies involving different races and patient‐specific factors are warranted. Additionally, the duration of interventions and follow‐ups was different and relatively short (within 6 months), with a lack of data on long‐term efficacy, while melanocyte regeneration may require a long time. As a result, our systematic review provides only preliminary conclusions on the short‐term effects of the combination therapy regimens for vitiligo. Finally, this meta‐analysis did not evaluate improvements in cost‐efficiency and quality of life, which are also important considerations in clinical decision‐making.

## 5 Conclusion

This systematic review and meta‐analysis demonstrated that fractional laser combined with PRP is an effective and safe therapy for adults suffering from vitiligo. For patients with stable vitiligo unresponsive to conventional therapies, this combination therapy may yield promising effects. Given its novelty, additional large‐scale, well‐designed RCTs are warranted in the future to validate these findings.

## Author Contributions

X.F., Y.D., and J.X. designed the research study. X.F., S.J., and X.Z. collected and analyzed the data. X.F. and J.X. wrote the paper and revisions.

## Ethics Statement

The authors have nothing to report.

## Consent

The authors have nothing to report.

## Conflicts of Interest

The authors declare no conflicts of interest.

## Data Availability

The data that support the findings of this study are available from the corresponding author upon reasonable request.
